# Constructing a MoS_2_ QDs/CdS Core/Shell Flowerlike Nanosphere Hierarchical Heterostructure for the Enhanced Stability and Photocatalytic Activity

**DOI:** 10.3390/molecules21020213

**Published:** 2016-02-15

**Authors:** Shijing Liang, Zhouming Zhou, Xiuqin Wu, Shuying Zhu, Jinhong Bi, Limin Zhou, Minghua Liu, Ling Wu

**Affiliations:** 1 State Key Laboratory of Photocatalysis on Energy and Environment, Fuzhou University, Fuzhou 350002, China; liangzhu-2015@outlook.com (S.Z.); bijinhong@fzu.edu.cn (J.B.); mhliu2000@fzu.edu.cn (M.L.); wuling@fzu.edu.cn (L.W.); 2 Department of Environmental Science and Engineering, College of Environment and Resource Fuzhou University, Minhou, Fujian 350108, China; fdzhouzming@163.com (Z.Z.); wxqbelieve@163.com (X.W.); 3 Department of Mechanical Engineering, The Hong Kong Polytechnic University, Hung Hom, Kowloon, Hong Kong, China; mmlmzhou@polyu.edu.hk

**Keywords:** MoS_2_ QDs, CdS-based composite, core-shell structure, photocatalysis, water splitting

## Abstract

MoS_2_ quantum dots (QDs)/CdS core/shell nanospheres with a hierarchical heterostructure have been prepared by a simple microwave hydrothermal method. The as-prepared samples are characterized by XRD, TEM, SEM, UV-VIS diffuse reflectance spectra (DRS) and N_2_-sorption in detail. The photocatalytic activities of the samples are evaluated by water splitting into hydrogen. Results show that the as-prepared MoS_2_ QDs/CdS core/shell nanospheres with a diameter of about 300 nm are composed of the shell of CdS nanorods and the core of MoS_2_ QDs. For the photocatalytic reaction, the samples exhibit a high stability of the photocatalytic activity and a much higher hydrogen evolution rate than the pure CdS, the composite prepared by a physical mixture, and the Pt-loaded CdS sample. In addition, the stability of CdS has also been greatly enhanced. The effect of the reaction time on the formations of nanospheres, the photoelectric properties and the photocatalytic activities of the samples has been investigated. Finally, a possible photocatalytic reaction process has also been proposed.

## 1. Introduction

In recent years, photocatalytic technology has been extensively used for producing H_2_ utilizing solar energy. However, typical TiO_2_ photocatalyst solely absorbs UV light, which is only about 4% of the entire solar spectrum. In view of the efficient utilization of visible light, developing suitable and novel photocatalysts, which work efficiently under a wide range of visible light irradiation conditions, is a very hot topic [1,2].

Among the various reported photocatalysts [3,4,5,6,7,8,9,10], metal sulfides are regarded as excellent candidates for visible light-driven photocatalysis because of their suitable band gap and high catalytic activity. In particular, CdS is the most famous photocatalyst with a band gap value of 2.4 eV, and its conduction band position is more negative than the H_2_O/H_2_ reduction potential [11,12]. However, there are several issues that still limit the photocatalytically-reductive activity of pure CdS particles. For example, CdS particles tend to aggregate and form larger particles, which result in a reduced surface area and a higher recombination rate of the photogenerated electron-hole pairs [11]. Another serious drawback of CdS photocatalysts is the problem of photocorrosion [13,14]. The sulfide anion can be easily oxidized by photogenerated holes. This photocorrosion effect leads to most CdS structures being highly unstable as photocatalysts and, thus, limits their practical application [15,16]. To solve these problems, many approaches have been proposed to enhance the photoreductive activity and photostability of CdS, like preparing quantized CdS nanocrystallites, designing controllable morphologies, depositing noble metals, preparing colloidal CdS and forming CdS-based composites. Numerous nanostructured CdS photocatalysts with controllable morphologies have been synthesized [17,18,19,20,21,22,23], such as nanospheres, nanorods, flowers, nanotubes, nanocubes, petals and nanobelts. All of these nanostructured CdS photocatalysts shows higher hydrogen production activity than the bulk CdS, but the problem of photocorrosion was still serious.

Cocatalysts can offer the low activation potentials for H_2_ or O_2_ evolution and act as active sites for H_2_ or O_2_ formation, so it is crucial for photocatalytic H_2_ production reactions [24,25,26,27,28]. Cocatalysts are capable of assisting in electron-hole separation at the cocatalyst/semiconductor interface; thus, it may significantly inhibit the photocorrosion effect. Among the developed low-cost cocatalysts, MoS_2_, which is composed of Mo atoms sandwiched between two layers of hexagonal closely-packed sulfur atoms, has been extensively investigated [29,30]. It is reported that MoS_2_ is an efficient co-catalyst when coupled with semiconductors, such as TiO_2_ [31,32], CdS [27] and g-C_3_N_4_ [28], having shown remarkable enhancement in H_2_ evolution and the degradation of pollutants. In contrast to the widely-used Pt particle co-catalyst, the various morphologies of MoS_2_ can be easily controlled by using hydrothermal methods or high temperature processes under H_2_S atmosphere. These MoS_2_ structures showed high co-catalytic activities for hydrogen evolution [32,33]. However, the obtained MoS_2_ nanostructures are usually formed of irregular aggregates of nanoparticles or stacked multilayers deposited on a substrate; this problem also inhibits the co-catalytic activities for hydrogen evolution. Therefore, up to now, our knowledge regarding how to design or fabricate efficient MoS_2_/CdS nanocomposites is far from satisfactory.

In this work, MoS_2_ quantum dots (QDs) were prepared by a photo-assisted chemical etching method for the first time, and then, noble metal-free flowerlike MoS_2_ QDs/CdS core/shell nanosphere photocatalysts were synthesized by a one-pot microwave hydrothermal route. The physicochemical properties of the as-prepared samples were characterized in detail. The possible formation of flowerlike nanospheres was also proposed. The photocatalytic activities of the samples were evaluated by water splitting into hydrogen under visible light irradiation. Furthermore, the probable influencing factors for the enhanced stability and activity of CdS have also been investigated.

## 2. Results and Discussion

### 2.1. Phase Structure and Morphology

**Figure 1:**
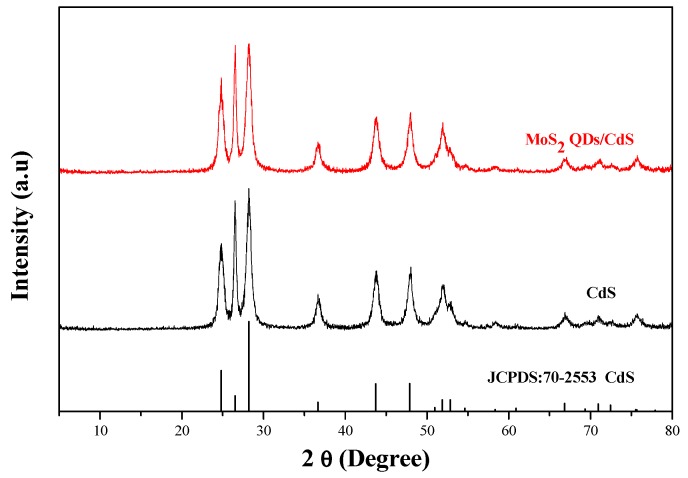
XRD patterns of the as-prepared CdS and MoS_2_ QDs-CdS.

The crystalline phase of MoS_2_ quantum dots (QDs) was analysis using the dried MoS_2_ QDs (Figure S1 in supplementary material). The diffraction peaks of the as-prepared MoS_2_ correspond well to the hexagonal phases of MoS_2_ (JCPDS Card No. 77-1716). The morphology of MoS_2_ QDs was determined by a TEM technique. It could be found that the sizes of MoS_2_ QDs were less than 10 nm and have a narrow size distribution with high dispersity in aqueous solution (Figure S2). Figure 1 shows the X-ray diffraction patterns of CdS and MoS_2_ QDs/CdS prepared by a microwave-hydrothermal reaction for 1 h. Diffraction peaks correspond well to the hexagonal phases of CdS (JCPDS Card No. 70-2553). Significantly, the XRD pattern of the MoS_2_ QDs/CdS composites shows that no impurity peaks could be detected from the XRD measurements. Notably, the diffraction peaks assigned to MoS_2_ are not observed over the MoS_2_ QDs/CdS sample; this is possible due to the low amount of MoS_2_ QDs loaded on CdS [34].

**Figure 2:**
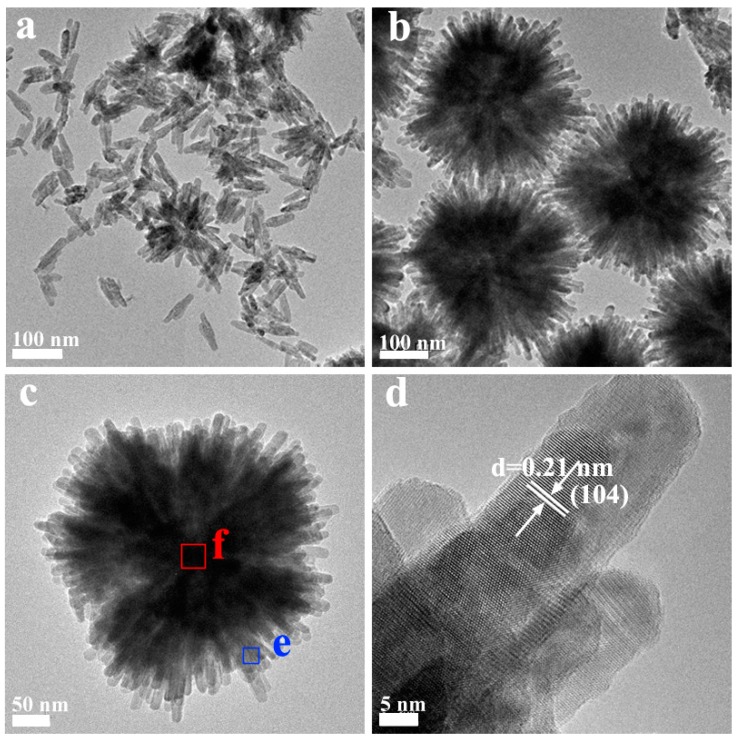

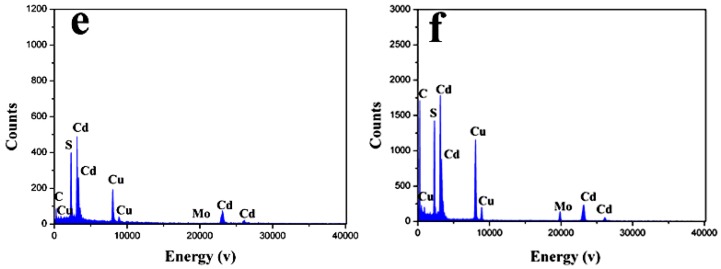
TEM images of pure CdS (**a**) and MoS_2_ QDs/CdS (**b**,**c**), HRTEM image of CdS MoS_2_ QDs/CdS (**d**) and EDS images (**e,f**) in the e and f areas of Figure 2c.

The samples were further characterized by TEM and SEM techniques to investigate the morphologies and the interfacial structures between MoS_2_ and CdS. As shown in Figure 2, the CdS prepared using l-cysteine as the S resource exhibits a monodispersed and uniform nanorod structure with a length of 100 nm and 10 nm in lateral size. The morphology is also confirmed by the SEM images, as shown in Figure S3. For the MoS_2_ QDs/CdS sample prepared for 1 h, it can be seen clearly that it shows a monodispersed and uniform flowerlike nanosphere structure with a size of 300 nm (Figure S4). From TEM images (Figure 2b,c), the nanosphere with hierarchical structures is constructed by many nanorods. The length of the nanorods is about 300 nm, and their width is about 20 nm. The HRTEM image shows the sample with high crystallinity. The space value of the clear lattice fringes (d = 0.21 nm) matches well with that of the (104) planes of the CdS crystal, indicating that the composition of the nanorod is CdS. Furthermore, we could also conclude that the nanorods grow along the [104] crystal plane.

Energy dispersive X-ray spectrometry (EDS) mapping analysis of the MoS_2_ QDs/CdS nanospheres was used to confirm the distribution of the Cd, Mo and S elements. As shown in Figure 2e, Cd and S elements were only found, but not Mo elements in the **e** area of Figure 2c. The Cu and C peaks in the spectra are derived from a carbon-coated copper TEM grid. However, in Figure 2f, Cd, S and Mo elements have been found in the **f** area of Figure 2c. Thus, it can be concluded that MoS_2_ is in the central portion of the nanoflower, but not on the nanorod. The MoS_2_ QDs/CdS nanospheres are a core/shell hetero-structure. MoS_2_ serves as the crystal nucleus for the growth of CdS nanorods. Notably, due to the direct growth of CdS nanorods on the MoS_2_, the interaction between MoS_2_ and CdS would be very strong. The excellent interfacial interaction between CdS and MoS_2_ is expected to improve the separation of photogenerated charge carriers, and therefore, the photocatalytic activity would be enhanced. The percentage of Mo element content is also evaluated to be about 0.1%.

### 2.2. The Possible Formation Process of the Flowerlike Nanospheres

**Figure 3:**
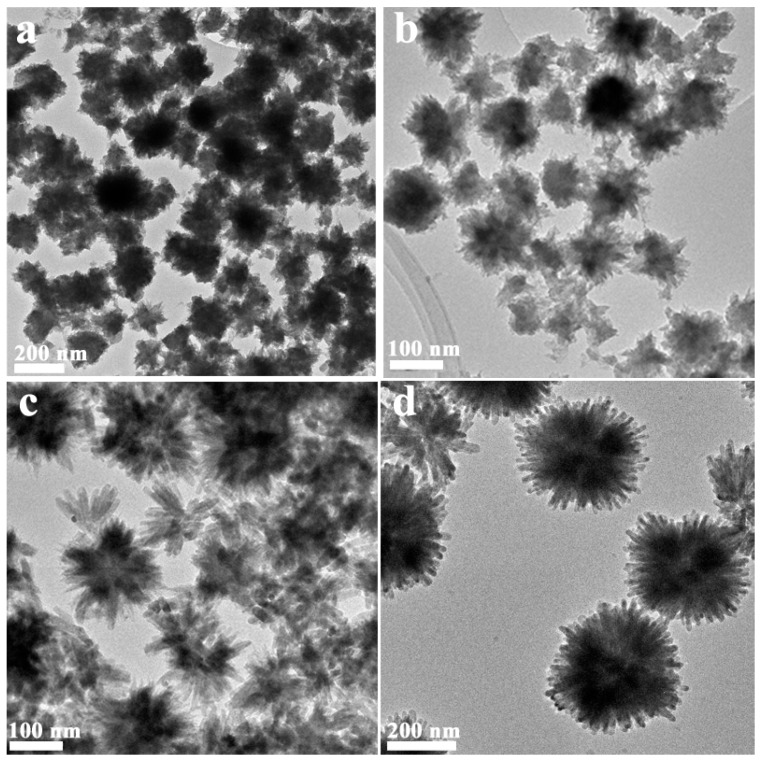
TEM images of the MoS_2_ QDs/CdS samples prepared for different times: (**a**) 5 min; (**b**) 15 min; (**c**) 30 min; (**d**) 60 min.

**Figure 4:**
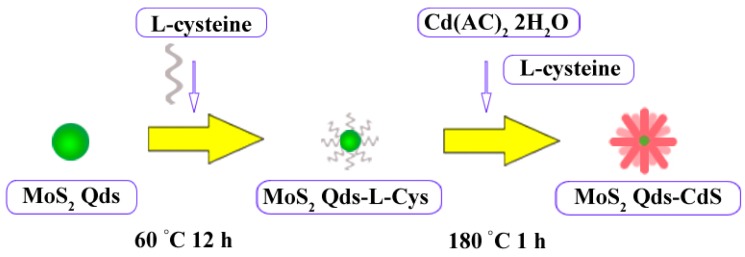
Schematic illustration of the formation process of the MoS_2_ QDs/CdS nanoflower.

In order to find the possible formation process of the nanospheres, the samples were also prepared by the microwave-hydrothermal reaction for 5 min, 15 min and 30 min at 180 °C. Figure 3a shows that the sample prepared for 5 min was constructed by the nanosphere and the irregular aggregates. From the XRD result (Figure S5), we could see that the sample is composed of crystalline CdS and intermediate products, although the crystallinity of the sample is poor. Prolonging the reaction time (Figure 3b,c), the part of the irregular aggregates is reduced, and the nanorods grow gradually. XRD result also shows the intermediate products disappear gradually, and the crystallinity of the sample is enhanced. When the samples were prepared for 1 h (Figure 3d), a monodispersed nanosphere composed of nanorods is obtained, and no irregular aggregates are observed. On the basis of the above experiment results, the morphological evolution mechanism of hierarchical flowerlike MoS_2_ QDs/CdS core/shell nanospheres can be proposed in following steps: (i) nucleation of irregular aggregates nanosphere and some intermediate products; (ii) dissolution-recrystallization growth; (iii) further growth and monodispersed nanospheres composed of nanorods are obtained. This may suggest that the reaction proceeds as a dissolution-recrystallization process [35,36,37]. As a comparison, MoS_2_ QDs-l-cys was replaced by MoS_2_ QDs during the microwave hydrothermal reaction process. It can be seen that flowerlike nanospheres were also formed. However, some large particles were also yielded, and the nanospheres exhibited a serious accumulation (Figure S6). That is, the MoS_2_ QDs play the role of the crystal nucleus for the growth of the nanosphere. l-cysteine not only serves as the S resource, but also facilitates the dispersion of nanosphere during the formation process. The possible formation process is schematically illustrated in Figure 4.

### 2.3. Photocatalytic Properties

**Figure 5:**
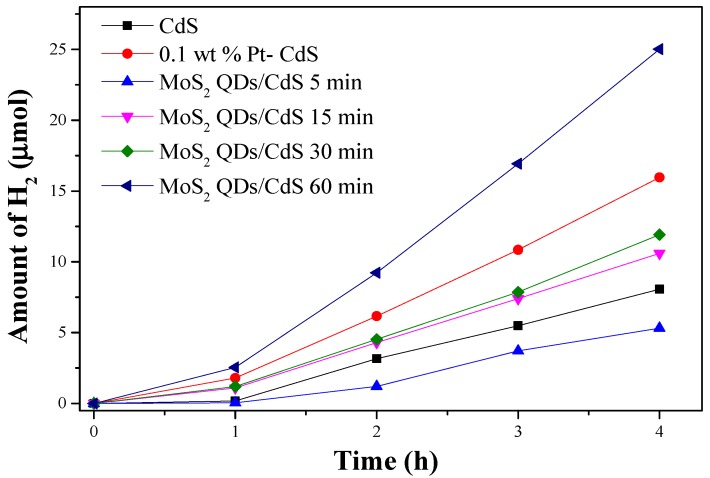
The amount of H_2_ evolution on CdS, MoS_2_ QDs/CdS (5 min,15 min,30 min 60 min), 0.1 wt % Pt-CdS catalysts under visible light irradiation (reaction conditions: 20 mg catalysts, 45 mL H_2_O containing 5 mL 0.02 M NaS and 0.025 M NaSO_3_, λ > 420 nm).

Figure 5 shows that the as-prepared samples exhibit a clear activity for the water splitting into H_2_. The amount of H_2_ evolution on pure CdS is 8.07 μmol after reaction for four hours, corresponding to about 100 μmol·h^−1^·g^−1^ of the H_2_ evolution rate. However, the activity of the MoS_2_ QDs/CdS sample could reach to 25.02 μmol after four hours and the H_2_ evolution rate is 312.75 μmol·h^−1^·g^−1^, which is over three-times higher than that of pure CdS. This may be owed to flowerlike hierarchical heterostructure morphology, and MoS_2_ QDs serve as the co-catalyst to facilitate the separation of photogenerated electron and hole pairs. The photocatalytic activities of MoS_2_ QDs/CdS samples are sensitive to the reaction time. With prolonging the reaction time, the activity is increased. The sample prepared for a short time may possess abundant defects to act at the recombination sites for the photogenerated electron and hole to reduce the activity. Furthermore, the 1D CdS nanorods could also promote the transfer of photogenerated electron and hole. This may be another reason for the enhanced photocatalytic activity. The MoS_2_ QDs/CdS sample prepared for 5 min exhibited the lowest H_2_ evolution rate (66.5 μmol·h^−1^·g^−1^), which may be due to the poor crystallinity and the existence of the intermediate products.

Pt was demonstrated to be the most promising cocatalyst for H_2_ evolution. As a comparison, the same content of Pt (0.1 wt %) was photodeposited on the as-prepared photocatalysts by directly dissolving H_2_PtCl_6_ (10 mg/L) into the reactant suspension. The activities of the CdS catalysts are increased from 100 to 199.5 µmol·h^−1^·g^−1^ after Pt loading. It should be pointed out that this H_2_ evolution rate is still lower than that of the corresponding MoS_2_ QDs/CdS sample. The results clearly indicate that MoS_2_ QDs could function as a more efficient co-catalyst for CdS photocatalyst compared to Pt. Furthermore, some controlled experiments have also been carried out. As shown in Figure S7, MoS_2_ QDs could not produce hydrogen under visible light irradiation. That is, the role of MoS_2_ QDs is as the cocatalyst. The sample (denoted as MoS_2_ QDs + CdS) was also prepared by a mechanical mixture of MoS_2_ QDs and CdS nanorods as a reference. It is found that MoS_2_ QDs + CdS exhibits a superior activity compared to pure CdS. However, the activity of this sample is much lower than that of the MoS_2_ QDs/CdS sample.

**Figure 6:**
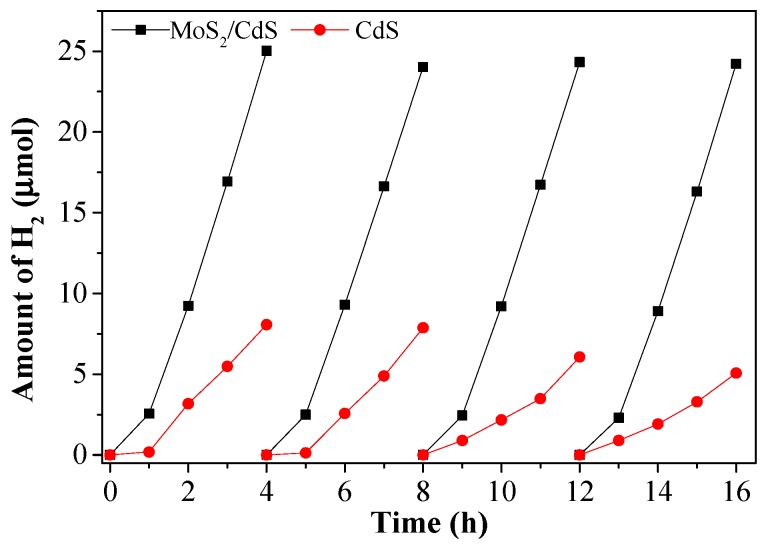
The stability of the activity over CdS and MoS_2_ QDs/CdS under visible light irradiation (reaction conditions: 20 mg catalysts, 45 mL H_2_O containing 5 mL 0.02 M NaS and 0.025 M NaSO_3_, λ > 420 nm).

The stability of the samples was also studied under visible light irradiation. As shown in Figure 6, no obvious loss activity has been observed over the MoS_2_ QDs/CdS sample. However, the hydrogen evolution rate of pure CdS shows a significant decrease during the photocatalytic water splitting process. All of the results indicate that MoS_2_ QDs could inhibit the photocorrosion of CdS.

### 2.4. Photoabsorption Performance and BET Surface Area

**Figure 7:**
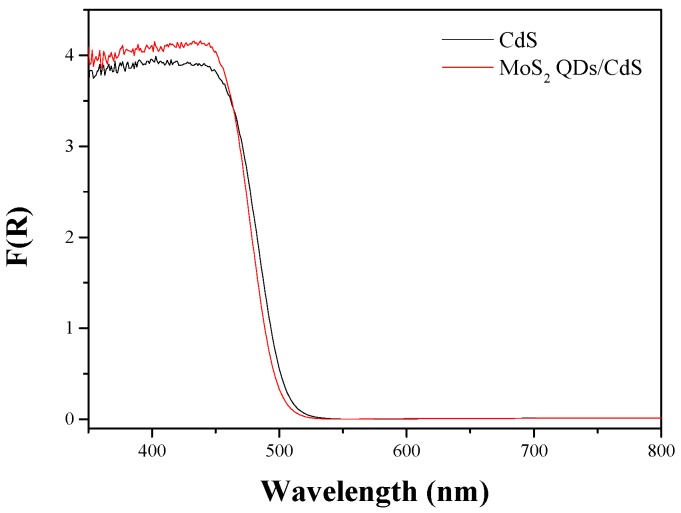
UV-VIS diffuse reflectance spectra (DRS) of CdS and MoS_2_ QDs/CdS.

As the photoabsorption properties play a crucial role in determining the photocatalytic activity, the UV-VIS diffuse reflectance spectra of the samples were recorded (Figure 7). Obviously, the samples have a strong absorption in the visible light region. The pure CdS sample has an absorption edge at 516 nm, corresponding to the energy value of 2.40 eV [38]. The absorption edge of MoS_2_ QDs/CdS has a small blue-shift, which is located at 510 nm. This small blue-shift may be due to the strong interaction between MoS_2_ and CdS. No clear absorption has been observed in the range of 550 to 800 nm, further indicating that no MoS_2_ QDs load on the shell of the nanosphere. It is worth noting that the absorption capacity of MoS_2_ QDs/CdS is higher than that of pure CdS in the range of 400 to 470 nm. The sample absorbing more photons with high energy will produce more electrons with a high reduction ability to reduce the water molecules. Thus, the sample may exhibit a high hydrogen evolution rate.

**Figure 8:**
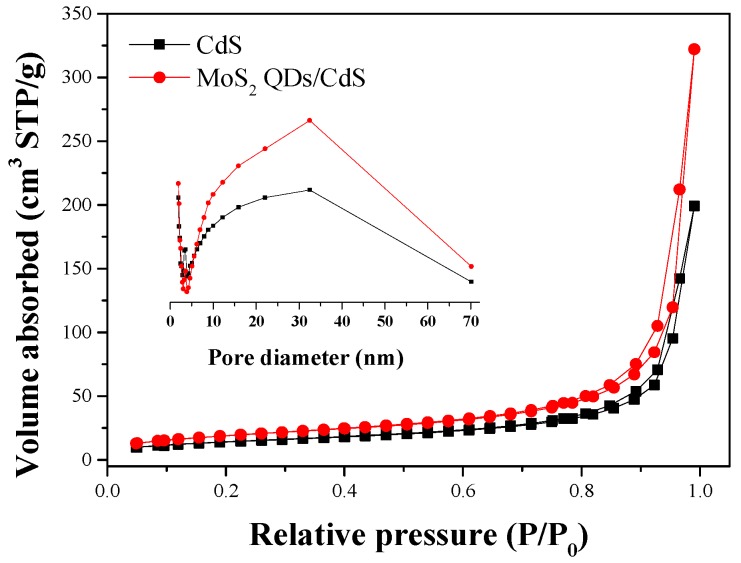
Nitrogen adsorption-desorption isotherms and the corresponding pore-size distribution curves (inset) for the CdS and MoS_2_ QDs/CdS.

The nitrogen adsorption-desorption isotherms of CdS and MoS_2_ Qds-CdS reveal that all of the samples exhibit a Type IV adsorption-desorption isotherm with a H_3_ hysteresis loop in the relative pressure range of 0.6 to 1.0 (Figure 8), indicating the presence of mesopores [38,39]. These mesopores may originate from the accumulation of CdS nanorods in the nanospheres. In addition, the BET specific surface area, pore volume, average pore size and H_2_ production rate of the samples are summarized in Table 1. The surface area, pore volume and average pore size of MoS_2_ QDs/CdS are larger than those of pure CdS. Generally, a larger BET surface area could provide more reaction sites for the photocatalytic reaction [40]. A large pore size and mesopores structures could facilitate the contact between the photocatalyst and reactants, as well as the mass transfer. Therefore, these may be one of the reasons for the enhanced H_2_ evolution activity.

**Table 1:** The BET surface area, pore volume, average pore size and H_2_ evolution rate of the samples.

Sample	Surface Area (m^2^·g^−1^)	Pore Volume (cm^3^·g^−1^)	Average Pore Size (nm)	H_2_ Evolution Rate (μmol·h^−1^·g^−1^)
CdS	50.7	10.3	25.13	100.8
MoS_2_ QDs/CdS	68.97	10.49	29.67	312.75

### 2.5. Photoelectrochemical Performance

**Figure 9:**
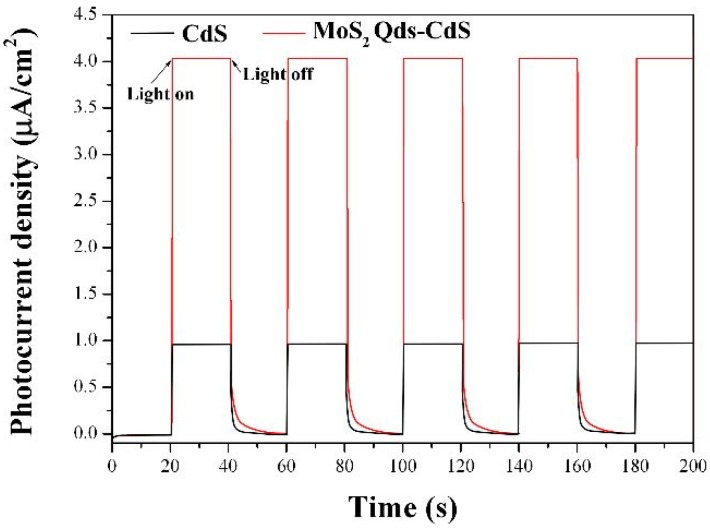
Transient photocurrent density *versus* time plotted for CdS and MoS_2_ QDs/CdS in 0.2 M Na_2_SO_4_ electrolyte under visible light irradiation.

To investigate the electron generation and the charge carrier transport characteristics of the as-prepared samples, the transient photocurrent responses of CdS and MoS_2_ QDs/CdS heterostructure electrodes were recorded over several on-off cycles under visible light irradiation. As shown in Figure 9, it could be found that the photocurrent value rapidly decreases to the initial value as soon as the irradiation of light was off, and the photocurrent comes back to a constant value when the light is on again. It could be seen that the CdS and MoS_2_ Qds-CdS electrodes have a steady photoelectrochemical performance under visible light irradiation (λ > 420 nm). Notably, the photocurrent intensity of MoS_2_ QDs/CdS is four-fold higher than that of the pure CdS. The results may show the MoS_2_ QDs/CdS sample with the higher e-h^+^ separation efficiency under visible light irradiation [41]. Therefore, a higher photocatalytic activity would be achieved. Moreover, the MoS_2_ Qds-CdS electrode photocurrent value decreased more slowly than the CdS electrode to the initial value when the irradiation of light is off, which suggests the low e-h^+^ recombination rate [42]. These photoelectrochemical performance results are in accord with the aforementioned photocatalytic H_2_ production activity.

### 2.6. Possible Photocatalytic Mechanism

**Figure 10:**
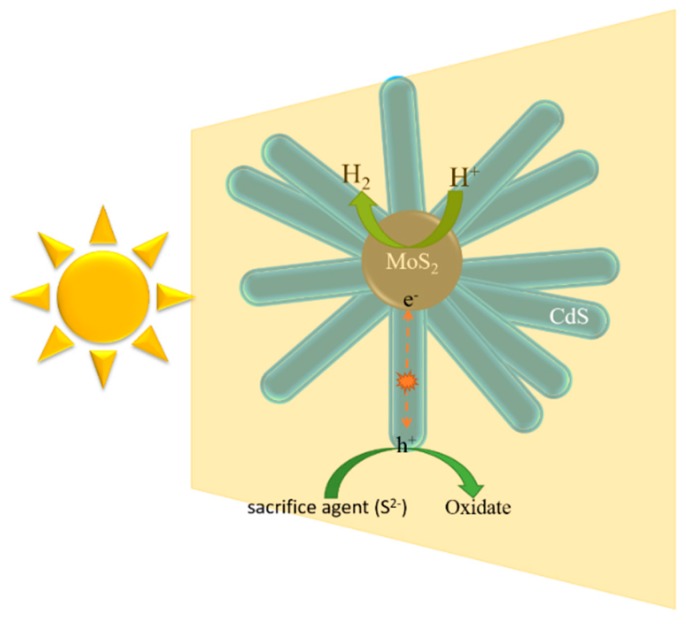
Schematic of photogenerated charge transfer and photocatalytic reaction over the MoS_2_ QDs/CdS under visible light irradiation.

On the basis of the results and discussions of the aforementioned experiments, the improved photocatalytic activities and inhibition of CdS corrosion of flowerlike MoS_2_ QDs/CdS composites may be attributed to the hierarchical heterostructure morphology, the fast charge separation and the slow charge carrier recombination. A probable mechanism for the photocatalytic hydrogen production process is proposed, as illustrated in Figure 10. Under visible light irradiation, the CdS nanorods are excited to generate charge carriers. The photogenerated electrons and holes of CdS can transfer quickly in the 1D nanorod structure to MoS_2_ QDs, owing to their intimate interfacial contact and matched band position. Due to the strong quantum confinement and edge effects of MoS_2_ QDs, they can act as an efficient cocatalyst to provide active sites with low overpotential for hydrogen production. For the other band, the hole will transfer to the other side to react with the sacrifice agent (S^2−^), which will suppress the corrosion of CdS. Furthermore, the mesostructure formed by the accumulation of nanorods could reflect the incident light multiple times, resulting in the improved light harvesting ability.

## 3. Experimental Section

### 3.1. Synthesis of Photocatalysts

All chemical reagents were of analytical grade and purchased from Sinopharm Chemical Reagent Co. Ltd. (Shanghai, China) without further purification.

(1) Synthesis of MoS_2_ QDs and MoS_2_ QDs-Lcys: 50 mg commercial MoS_2_ powder and 3 mL hydrogen peroxide (H_2_O_2_, 30 wt %) were added into 100 mL DI-water in a quartz tube under vigorous stirring at room temperature to ensure that the solution was fully mixed. Then, a light-grey suspension was obtained through UV light irradiation for 3 h. The resultant supernatant was centrifuged at 10,000 rpm for 15 min to separate the bulk MoS_2_. Finally, the MoS_2_ QDS suspension was formed. For the preparation of the MoS_2_ QDs-Lcys precursor, 40 mg l-cysteine were added into 10 mL of the as-prepared MoS_2_ QDS suspension under vigorous stirring.

(2) Synthesis of MoS_2_ Qds–CdS: 1 mL of the MoS_2_ Qds-Lcys suspension, 0.27 g of Cd(Ac)_2_·2H_2_O and 0.24 g of l-cysteine were dissolved in 30 mL of ethanolamine-water solution (volume ratio = 1:1) under magnetic stirring for 1 h. l-cysteine served as the S resource. The solution was then transferred into a Teflon-lined autoclave, which was placed in a microwave-hydrothermal synthesis system (ETHOS One, Milestone, Sorisole, Italy) and kept at 180 °C for a given time. After the reaction, the autoclave was cooled to room temperature, and the resulting precipitate was collected by centrifugation, alternately rinsed several times with distilled water and ethanol. The final product was dried in a vacuum oven at 80 °C over 12 h. The referenced CdS was also synthesized following the same process without the MoS_2_ QDs suspension.

### 3.2. Characterization

The as-prepared samples were characterized by powder X-ray diffraction (XRD) on a Bruker D8 Advance X-ray diffractometer (Karlsruhe, Germany) operated at 40 kV and 40 mA with Ni-filtered Cu K_α_ irradiation (λ = 1.5406Å). Transmission electron microscopy (TEM) images were recorded using a JEOL model JEM 2010 EX microscope (Peabody, MA, USA) at an accelerating voltage of 200 kV. Scanning electron microscopy (SEM) images were obtained with a Nova NanoSEM 230 microscopy (FEI Corp., Hillsboro, OR, USA) UV-VIS diffuse reflectance spectra (UV-VIS DRS) were obtained by using a UV-VIS spectrophotometer (Varian Cary 500, Santa Clara, CA, USA), and the data were converted to Kubelka-Munk (KM) functions. Barium sulfate was used as a reference. The Brunauer-Emmett-Teller (BET) surface area was measured with an ASAP2020M apparatus (Micromeritics Instrument Corp., Norcross, GA, USA).

### 3.3. Photoelectrochemical Measurements

The working electrode was prepared on fluorine-doped tin oxide (FTO) glass, which was cleaned by sonication in chloroform, acetone and ethanol for 30 min. The glass was then rinsed with pure water (18 MΩ cm) and dried in the air. The FTO slide was dip coated with 10 μL of slurry, which was obtained from mixture of 5 mg powder and 0.5 mL dimethylformamide under sonication for 2 h to get a thin film of the samples coated on the FTO slide. After air drying naturally, a copper wire was connected to the side part of the FTO glass using conductive tape. The uncoated parts of the electrode were isolated with an epoxy resin, and the exposed area of the electrode was 0.25 cm^2^. The electrochemical measurements were performed in a conventional three-electrode cell, using a Pt plate and a saturated Ag/AgCl electrode as the counter electrode and reference electrode, respectively. The working electrodes were immersed in a 0.2 M Na_2_SO_4_ aqueous solution without any additive for 30 s before measurement. The photocurrent measurements were conducted with a CHI650E electrochemical workstation (Chenhua Instruments, Shanghai, China). A 300-W Xe lamp (Beijing Trustech, PLS-SXE300c, Beijing, China) with a 420-nm cut-off filter was used as a light source.

### 3.4. Photocatalytic Activity

The photocatalytic activities of MoS_2_/CdS core/shell composites were evaluated by the decomposition of H_2_O in an aqueous solution. The catalyst (20 mg) was suspended in a 45-mL Pyrex glass vessel, which contained 5 mL 0.02M NaS and 0.025 M NaSO_3_ solutions. The light source was a 300-W Xe lamp (Beijing Trustech, PLS-SXE300c,) with a 420-nm cut-off filter. The temperature of the reactant solution was maintained at 275 K by a flow of cooling water during the reaction. The reaction solution was evacuated several times to remove air completely prior to irradiation. The amount of H_2_ produced was analyzed using an online gas chromatography. Zero-point-one weight percent of Pt cocatalyst was photodeposited on the catalysts by directly dissolving H_2_PtCl_6_ (10 mg/L) into the reactant suspension, if necessary.

## 4. Conclusions

In conclusion, monodispersed MoS_2_ QDs/CdS core/shell flowerlike nanospheres have been prepared by a microwave hydrothermal method. The as-prepared MoS_2_ QDs/CdS samples exhibit superior photocatalytic activity compared to pure CdS, Pt/CdS and the composite prepared by a physical mixture. The hydrogen evolution rate of MoS_2_ QDs/CdS could reach 312.75 µmol·h^−1^·g^−1^, which is over three-times that of CdS. Furthermore, the stability of CdS is greatly enhanced. The enhanced activity of CdS could be attributed to the unique morphology, the improved charge separation rate and the reduced charge recombination rate. Notably, MoS_2_ QDs is more suitable as a cocatalyst for hydrogen evolution on CdS than the noble metal Pt in our experiments.

## Supplementary Materials

Supplementary materials can be accessed at: http://www.mdpi.com/ 1420-3049/21/2/213/s1.
