# Supplementary material for: Constructing a MoS2 QDs/CdS Core/Shell Flowerlike Nanosphere Hierarchical Heterostructure for the Enhanced Stability and Photocatalytic Activity
Source: Molecules. 2016 Feb 15;21(2):213. doi: 10.3390/molecules21020213 (PMC6273490; doi:10.3390/molecules21020213)
Supplement: Supplementary file 1 [file molecules-21-00213-s001.pdf]

Supplementary Materials: Constructing a MoS_2 QDs/CdS Core/Shell Flowerlike Nanosphere Hierarchical Heterostructure for the Enhanced Stability and Photocatalytic Activity

Shijing Liang, Zhouming Zhou, Xiuqin Wu, Shuying Zhu, Jinhong Bi, Limin Zhou, Minghua Liu and Ling Wu

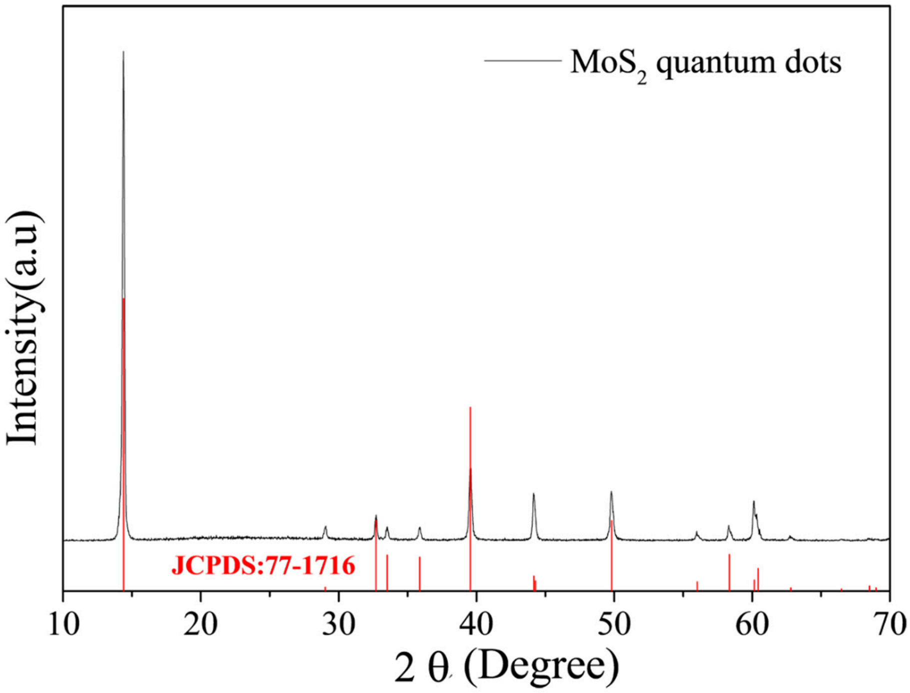

Figure S1. XRD pattern of MoS_2 quantum dots.

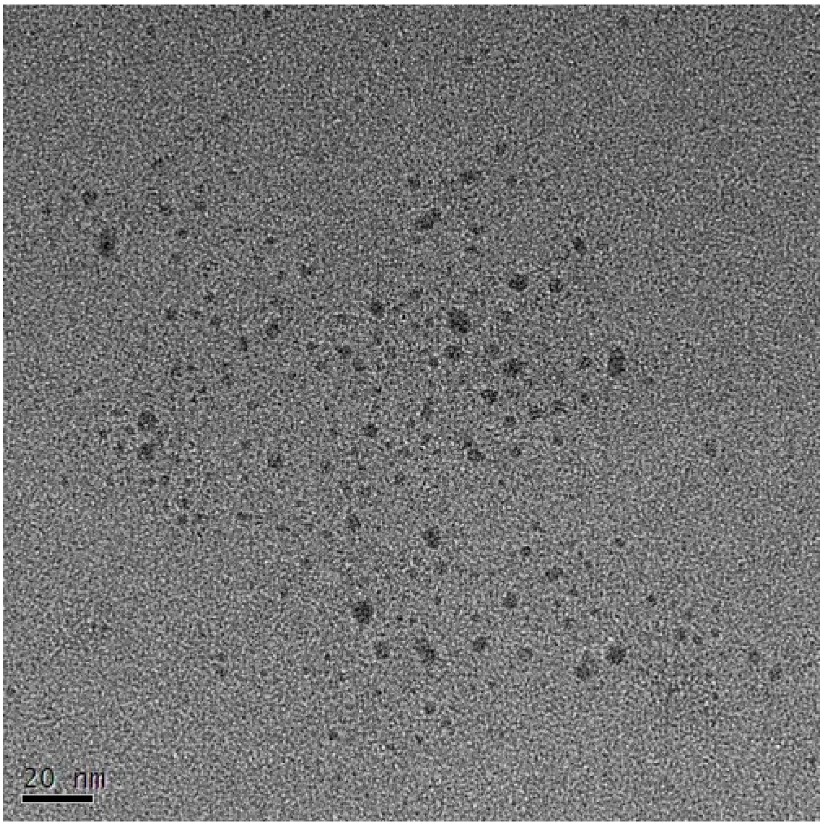

Figure S2. TEM image of MoS_2 quantum dots.

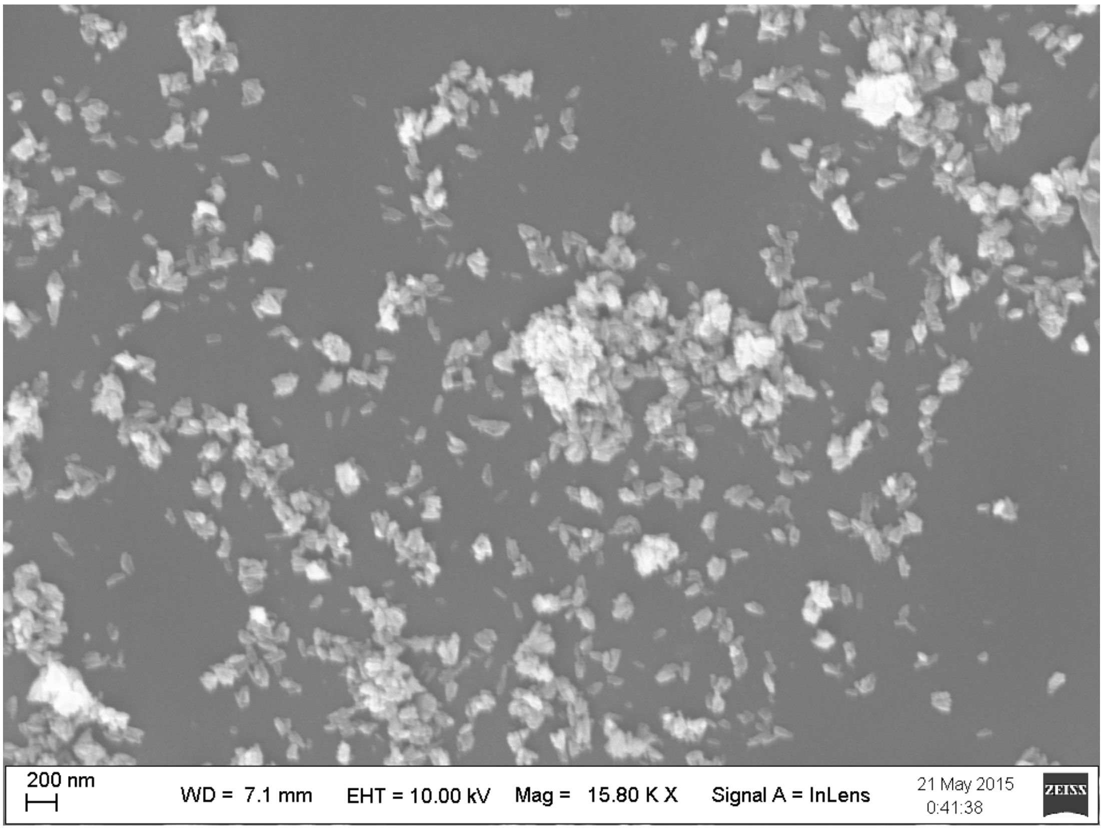

Figure S3. SEM image of the pure CdS sample.

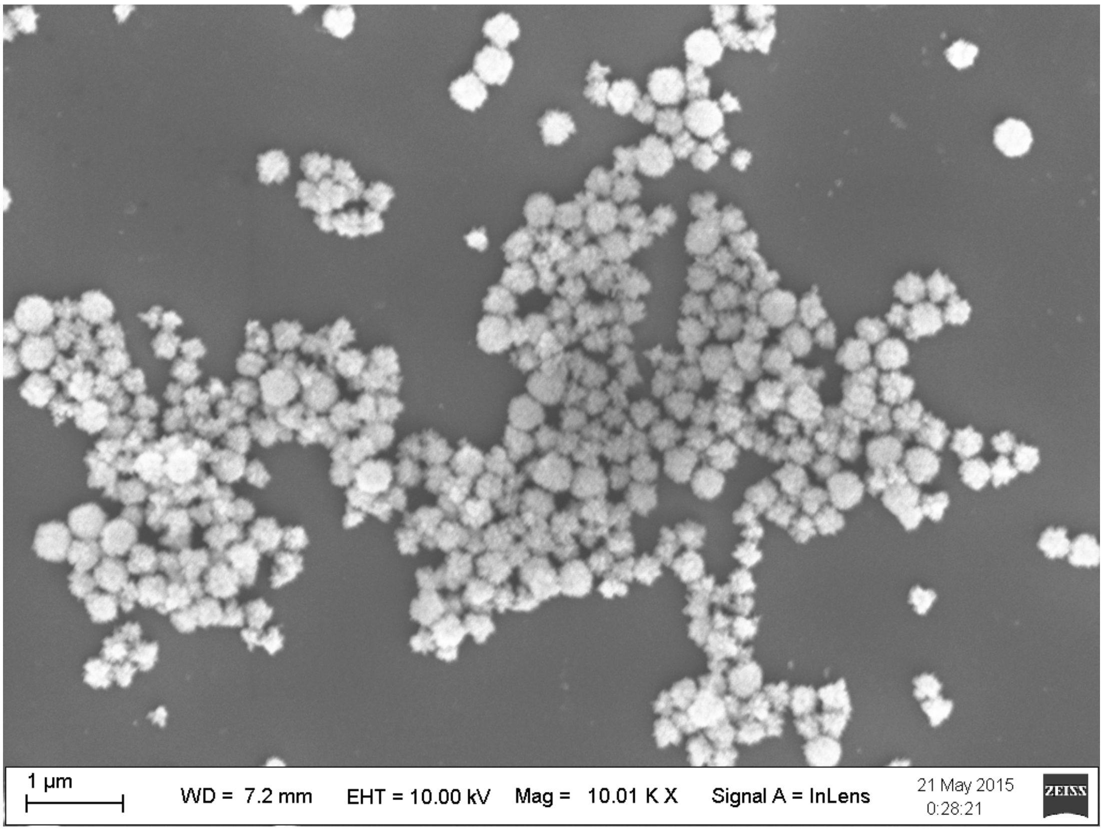

Figure S4. SEM image of the MoS_2 Qds/CdS sample.

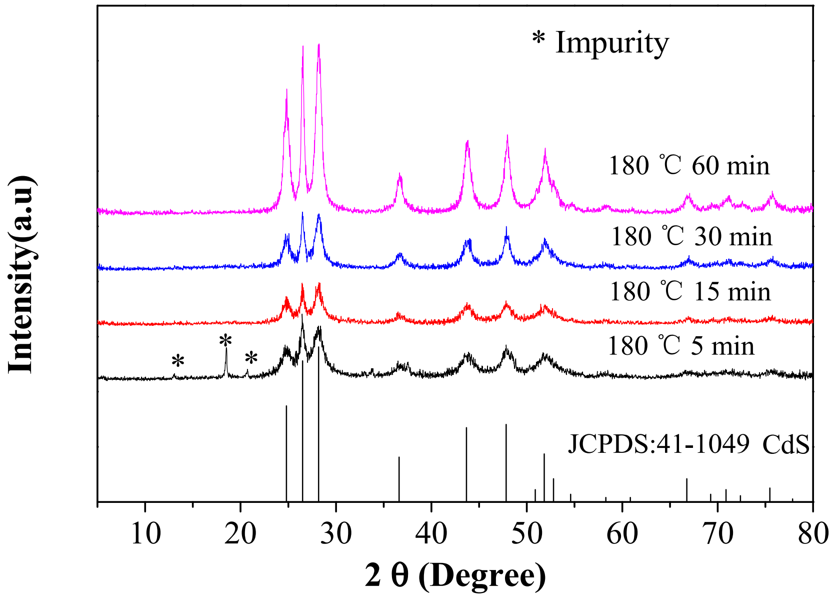

Figure S5. XRD patterns of the MoS_2 QDs/CdS samples prepared for different times.

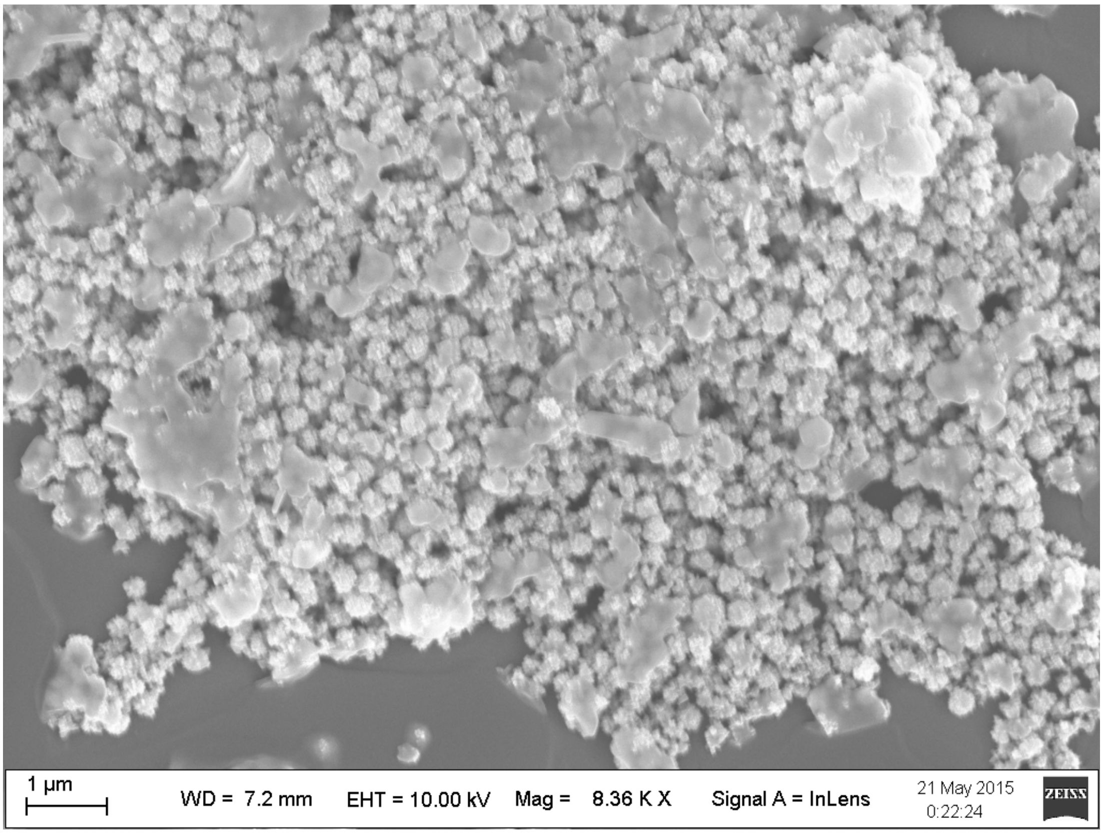

Figure S6. SEM image of MoS_2 Qds-CdS, MoS_2-Qds-L-cys was replaced with MoS_2-Qds in the microwave hydrothermal route.

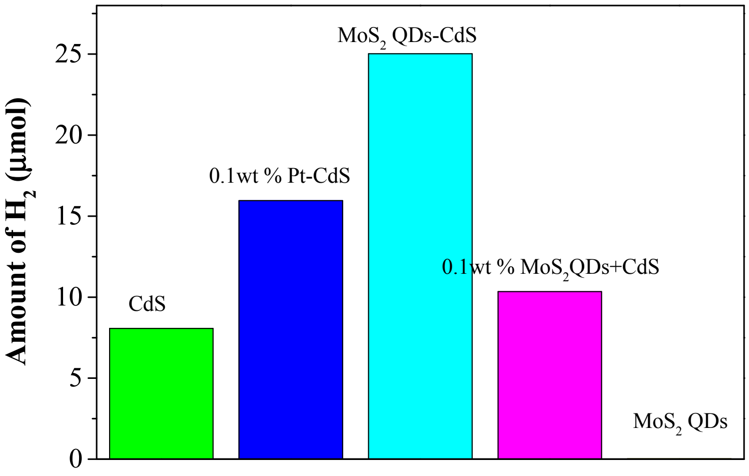

Figure S7. The amount of H_2 evolution over CdS, 0.1 wt% Pt/CdS, MoS_2 QDs/CdS, 0.1 wt% MoS_2 QDs + CdS and MoS_2 QDs under visible light irradiation after 4 h.
